# Combined Use of Laparoscopy and an Open Inguinal Approach for Repair of a De Garengeot Hernia

**DOI:** 10.7759/cureus.58771

**Published:** 2024-04-22

**Authors:** Bakhtawar Mushtaq, Gianfranco Galantini, Jesse Ottaway, Urooj Khalid, Robert Myers, Gabrielle Perrotti, Danelle Bertozzi

**Affiliations:** 1 General Surgery, Abington Jefferson Hospital, Philadelphia, USA

**Keywords:** appendectomy, laparoscopic, appendicitis, femoral hernia, incarcerated

## Abstract

Femoral hernias carry an increased risk of incarceration. De Garengeot hernia, a rare subset, occurs when the appendix herniates through the femoral canal. Due to its rarity, various surgical approaches have been explored, including isolated groin incisions, combined approaches, and exclusive laparoscopic interventions. This case involved a 58-year-old female diagnosed with a De Garengeot hernia and nonperforated acute appendicitis, managed through a combined laparoscopic and an inguinal approach, and underwent laparoscopic appendectomy and open repair of femoral hernia using a biologic mesh. In this case, the combined approaches facilitated a successful hernia repair and appendectomy while enabling a swift recovery. This case highlights the effectiveness of the combined minimally invasive and inguinal approach in optimizing outcomes for patients with De Garengeot hernia.

## Introduction

Femoral hernias account for 3% of groin hernias, are more common in females, and are at higher risk for incarceration secondary to a narrow femoral neck [1]. In 1731, Rene Jacques Croissant De Garengeot, a French surgeon, described a rare (0.08-0.13%) condition in which an appendix travels through the femoral canal and presents as an incarcerated femoral hernia [2]. Due to its rarity, patients with De Garengeot hernia have been treated with multiple surgical approaches including isolated groin incisions, combined groin incision and laparotomy, laparoscopic hernia repair with laparoscopic appendectomy, and less commonly a groin incision and laparoscopic appendectomy [3]. It may present as a bulge below the inguinal ligament and is frequently associated with pain. Here we present a case where a De Garengeot hernia was managed using a combined laparoscopic and inguinal approach that allowed for a successful repair with expedient postoperative recovery.

## Case presentation

**Figure 1:**
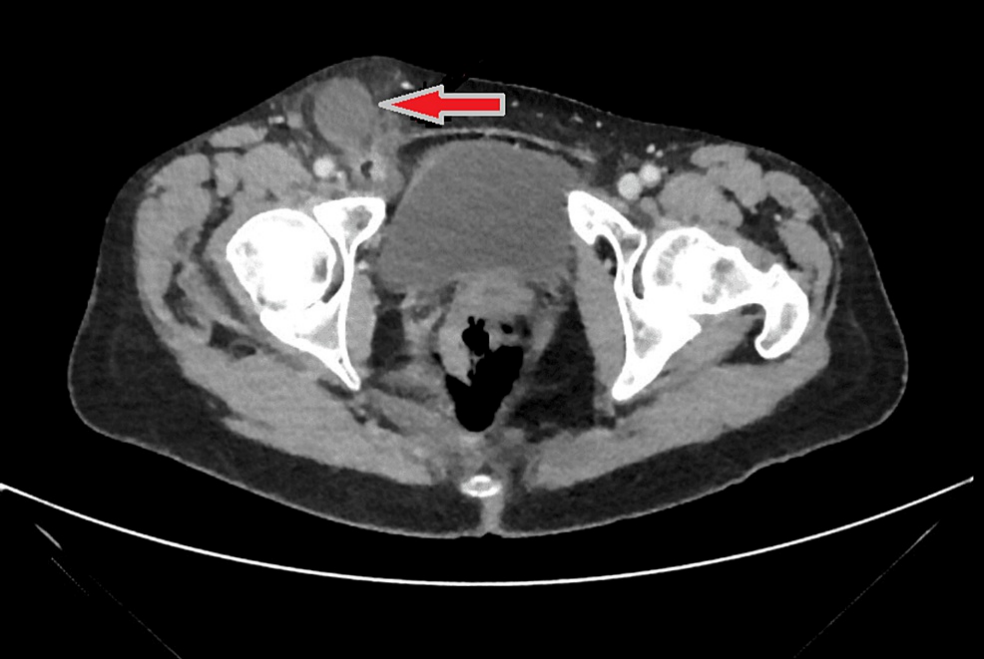
CT abdomen pelvis indicated possible perforated acute appendicitis with 3.2x4.3 cm fluid collection (appendiceal tip abscess) within a femoral hernia CT, computed tomography

A 58-year-old female with a past medical history of presumed right inguinal hernia presented with one day of pre-syncopal symptoms. She noticed a painless bulge in her right groin for three days. Her last bowel movement was on the morning of the presentation. Her abdomen was soft, non-tender, and had no guarding. On exam, there was a non-reducible, non-tender bulge in the right groin without skin changes. She was afebrile and tachycardic. Her white blood count (WBC) was 11 g/dL and lactate was 2.4 mmol/L. A computed tomographic (CT) scan of the abdomen/pelvis indicated a possible perforated acute appendicitis with a 3.2x4.3 cm fluid collection (appendiceal tip abscess) within a femoral hernia (Figure 1).

**Figure 2:**
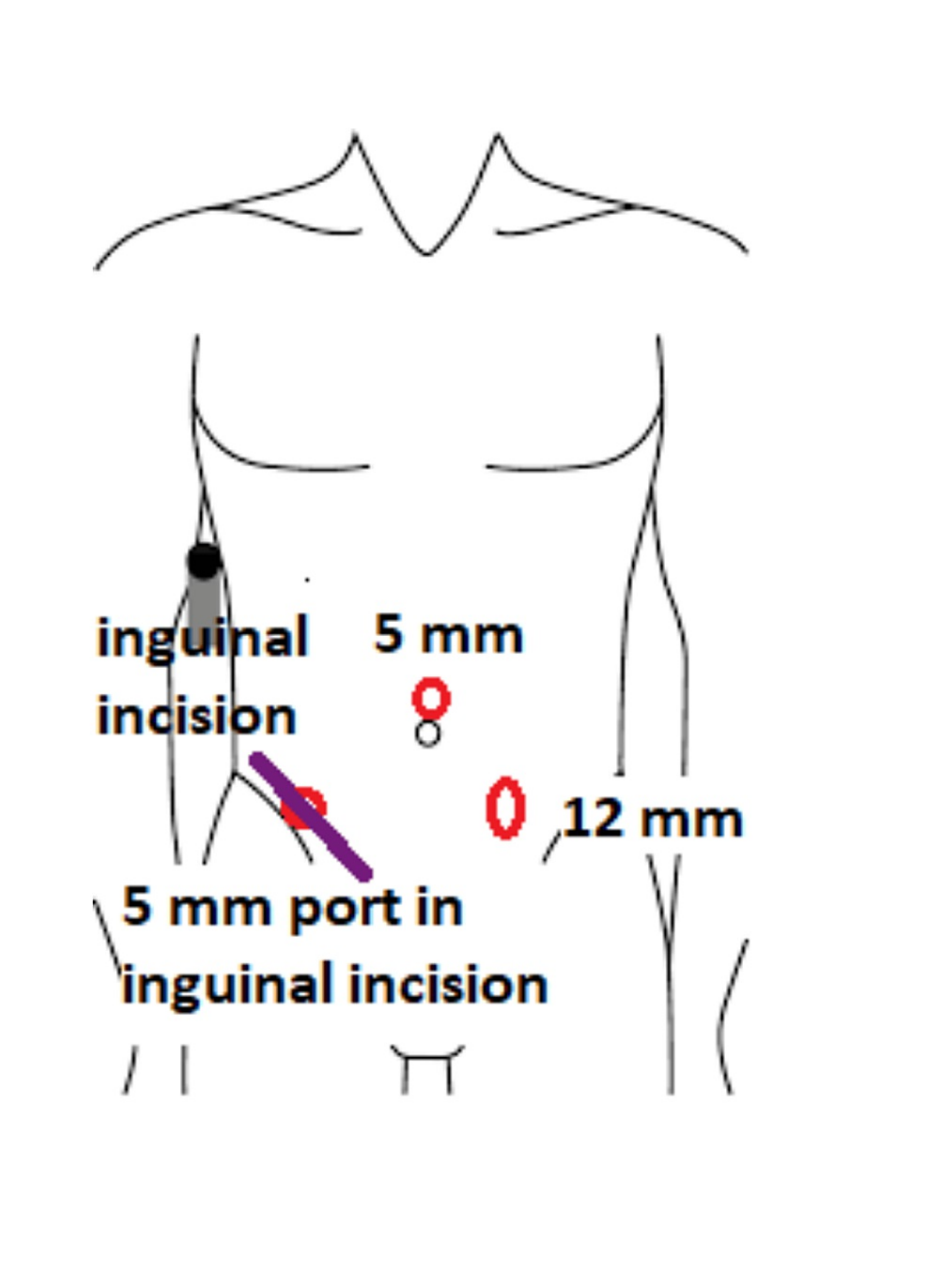
Schematic diagram showing port placement This image was created by the authors of this manuscript.

She received intravenous cefepime and flagyl and was taken to the operating room. Under general anesthesia, the femoral sac was accessed through a standard oblique incision above the inguinal ligament. Upon initial inspection, the appendiceal tip was hemorrhagic. The small hernia defect made it difficult to deliver the proximal appendix; therefore, a laparoscopic approach was incorporated to complete the rest of the procedure (Figure 2).

A 5 mm periumbilical port was placed superior to the umbilicus, and pneumoperitoneum was established. A 12 mm port was placed in the left lower quadrant. The laparoscopic approach allowed the appendix to be reduced back into the abdomen. A 5 mm port was placed through the femoral canal. The appendiceal tip was inflamed but nonperforated.** **The base of the appendix and mesentery were divided using the stapler. The appendix was removed through the left lower quadrant incision. In the absence of an appendiceal perforation, the hernia defect was closed using an absorbable mesh plug. The patient tolerated a regular diet and was discharged home on postoperative day one. Pathology was significant for benign appendix with peri-appendiceal fat inflammation.

## Discussion

Femoral hernias represent a relatively uncommon subset of groin hernias, comprising approximately 3% of cases, with a higher prevalence in females. The increased incidence in females is thought to be attributed to anatomical factors such as a wider pelvis and femoral ring. Femoral hernias are particularly predisposed to incarceration due to the narrow femoral neck, which can lead to strangulation of the herniated contents. 

Herniation of the appendix in the femoral canal is usually attributed to an abnormal pelvic location during embryologic development [4]. De Garengeot hernia is traditionally associated with acute appendicitis leading to incarceration or strangulation given the narrow femoral ring [4]. Similar to an inguinal hernia, it also presents as a painful and irreducible groin lump [5]. These nonspecific symptoms pose challenges to accurate diagnosis. The gold standard for diagnosis remains CT imaging [1]. Multiple case studies have shown various surgical approaches to a De Garengeot hernia that were individualized based on the patient's presentation. These include but are not limited to isolated groin incisions (Lockwood-low incision, Lotheissen transinguinal incision, and McEvedy high incision), combined groin incisions with laparotomy, laparoscopic hernia repair with appendectomy, and less commonly, groin incisions with laparoscopic appendectomy [3].

In this case, a combined laparoscopic and inguinal approach allowed for the successful management of De Garengeot hernia and facilitated the patient's expedited recovery and discharge. This approach was essential to reduce the hernia as the proximal appendix could not be delivered through the femoral ring to allow for a groin-only incision. Instead of creating a third port incision, the femoral hernia defect served as the port site. Following the appendectomy, the appendix may then be removed in a standard fashion via the 12 mm port. Closure of the femoral may be achieved using a mesh plug, provided there is no concern about appendiceal perforation and contamination of the surgical field [6]. In events of appendiceal perforation, standard McVay repair is recommended, as placement of mesh is associated with an increased risk of surgical site infections [5]. 

## Conclusions

De Garengeot hernia remains a rare presentation of femoral hernia, posing a challenge to diagnosis and treatment. Utilization of cross-sectional imaging in the modern age aids in the pre-operative diagnosis. Incarceration or strangulation of the appendix makes surgical management challenging. Given no standardized repair technique, knowledge of the entity, clinical presentation, complications associated with delay in treatment, and available surgical options are crucial for timely intervention. The utilization of hybrid surgical techniques, when possible, represents a valuable strategy for achieving optimal outcomes while facilitating a fast postoperative recovery.
